# Low birthweight and preterm birth: trends and inequalities in four population-based birth cohorts in Pelotas, Brazil, 1982–2015

**DOI:** 10.1093/ije/dyy106

**Published:** 2018-06-22

**Authors:** Mariangela F Silveira, Cesar G Victora, Bernardo L Horta, Bruna G C da Silva, Alicia Matijasevich, Fernando C Barros, Pelotas Cohorts Study Group, Aluisio J D Barros, Ana M B Menezes, Andrea Dâmaso Bertoldi, Diego G Bassani, Fernando C Wehrmeister, Helen Gonçalves, Iná S Santos, Joseph Murray, Luciana Tovo-Rodrigues, Maria Cecilia F Assunção, Marlos Rodrigues Domingues, Pedro R C Hallal

**Affiliations:** 1 Federal University of Pelotas, Brazil; 2 University of Toronto, Canada; 1 Maternal and Child Health Department, Federal University of Pelotas, Pelotas, Brazil; 2 Postgraduate Program in Epidemiology, Federal University of Pelotas, Pelotas, Brazil; 3 Department of Preventive Medicine, Faculty of Medicine FMUSP, University of São Paulo, São Paulo, Brazil; 4 Postgraduate Program in Health and Behavior, Catholic University of Pelotas, Pelotas, Brazil

**Keywords:** Infant, low birthweight, premature birth, cohort studies, birthweights, preterm births

## Abstract

**Background:**

Despite positive changes in most maternal risk factors in Brazil, previous studies did not show reductions in preterm birth and low birthweight. We analysed trends and inequalities in these outcomes over a 33-year period in a Brazilian city.

**Methods:**

Four population-based birth cohort studies were carried out in the city of Pelotas in 1982, 1993, 2004 and 2015, with samples ranging from 4231 to 5914 liveborn children. Low birthweight (LBW) was defined as <2500 g, and preterm birth as less than 37 weeks of gestation. Information was collected on family income, maternal skin colour and other risk factors for low birthweight. Multivariable linear regression was used to estimate the contribution of risk factors to time trends in birthweight.

**Results:**

Preterm births increased from 5.8% (1982) to 13.8% (2015), and LBW prevalence increased from 9.0% to 10.1%, being higher for boys and for children born to mothers with low income and brown or black skin colour. Mean birthweight remained stable, around 3200 g, but increased from 3058 to 3146 g in the poorest quintile and decreased from 3307 to 3227 g in the richest quintile. After adjustment for risk factors for LBW, mean birthweight was estimated to have declined by 160 g over 1982–2015 (reductions of 103 g in the poorest and 213 g in the richest quintiles).

**Conclusions:**

Data from four birth cohorts show that preterm births increased markedly. Mean birthweights remained stable over a 33-year period. Increased prevalence of preterm and early term births, associated with high levels of obstetric interventions, has offset the expected improvements due to reduction in risk factors for low birthweight.

## Introduction

Preterm birth and low birthweight are major risks factor for neonatal, infant and under-five mortality. Globally, 16% of all children are born with low birthweight (LBW, <2500 g),^1^ and 11.1% are born preterm (less than 37 completed weeks of gestation).^2^ In Brazil, data from the National Live Births System (SINASC) which covers 96% of all births in the country, show a prevalence of preterm birth of 11.4% in 2016.^3^ Although several countries fail to report on birthweight, it is estimated that 9% of Latin American newborns fall in this category.^1^

LBW may result from preterm delivery, intrauterine growth restriction or a combination of both. A systematic review found that each of these conditions contributes to approximately half of all LBW babies.^4^ In 2010, preterm births (PTB) were estimated to account for 11% of all live births in the world.^2^ PTB complications are the leading cause of death among children under 5 years of age, being responsible for approximately 1 million deaths in 2015.^5^ PTB is also associated with long-term consequences including cerebral palsy, sensory deficits, learning disabilities and respiratory illnesses, compared with term birth.^2^

Fetal or intrauterine growth restriction (IUGR, defined as a birthweight below the 10th centile of a reference population) was estimated to affect 19.3% of all babies born in low- and middle-income countries in 2012.^6^ IUGR is associated with a near 2-fold increase in the risk of neonatal and post neonatal mortality among term infants; the risk is markedly higher for newborns who present both IURG and PTB.^7^ IUGR is also a major contributor to child undernutrition and to poor psychomotor development.^7^

In 2008, we reported on trends in birthweight and preterm births in three population-based birth cohorts in the city of Pelotas in 1982, 1993 and 2004.^8^ We now extend this time-series to incorporate the 2015 birth cohort. Our objectives were to report on time trends in preterm births, LBW and mean birthweight, and to assess inequalities in birthweight according to family income, maternal skin colour and sex of the child.

## Methods

Over the course of the years of 1982, 1993, 2004 and 2015, all hospital births in the city of Pelotas were identified through daily visits to all maternity wards, and mothers who lived in the urban area were invited to join the studies. Those who accepted were interviewed by the study team using a structured questionnaire, and anthropometric data were obtained from the women and their newborns. Methodological details of each cohort (1982, 1993, 2004 and 2015) are provided elsewhere.^9–12^

Newborns were weighed within 24 h of birth, using paediatric scales with a precision of 10 g, in each participating hospital. LBW was defined as a birthweight below 2 500 g.^13^

The method of assessment of gestational age changed over time. In 1982 and 1993 it was based on the date of the last menstrual period (LMP) provided by the mother, and unknown and unreliable cases were excluded. These represented 21.0% and 10.2% of all newborns, respectively. In 2004 and 2015, we adopted the best obstetric estimate based primarily on first- or second-trimester ultrasound when available. When ultrasound was not available, the estimate was based on the LMP. For the 2004 and 2015 cohorts, we also present estimates based solely on the LMP for comparability with the earlier cohorts.

Mothers were interviewed during the hospital stay and provided information on monthly family income, calculated from the sum of incomes of family members, and divided into quintiles. As noted in a previous publication,^14^ income data for 1993 are less reliable than for the other cohorts, due to hyperinflation during that year. Information was also collected on maternal skin colour, categorized as white, black or other by the interviewer, except in 2015 when it was self-reported; in 1982, only two categories (white or other) were recorded.

For the multivariable analyses, information was also collected on known risk factors for birthweight which were measured in the four cohorts, including maternal age (<20, 20–24, 25–29, 30–34, >=35 years), schooling (<4, 5–8, 9–11 or >=12 years), height (<150, 150–159, 160–169, >=170 cm), pre-pregnancy body mass index (<18.5, 18.5–24.9, 25.0–29.0, >= 30.0 kg/m^2^), smoking during pregnancy (yes, no), parity (0, 1, >=2 children), preceding birth interval (<24 months, >=24 months, primipara), antenatal care (<4, 4–7, >=8 visits) and marital status (married or in union, other). In the multivariable analyses, income was measured in minimum wages at the time of each cohort, and coded as <=1.0, 1.1–3.0, 3.1–6.0, 6.1–10.0 and >10. Details on the instruments used to collect these variables are available in other articles in this issue.^14–16^ Maternal reports of diabetes and hypertension during pregnancy were included in the multivariable analyses.

For family income expressed in quintiles, the slope index of inequality (SII) and concentration index (CIX) were calculated to assess absolute and relative inequalities. The SII can be interpreted as the difference, in percentage points, between prevalence at the top and at the bottom of the income scale; it ranges from -100% to 100% points. The concentration index is a measure similar to the Gini coefficient; a value of zero indicates perfect equality, whereas negative values indicate higher prevalence of the outcome among the poor.^17^^,^^18^

All analyses were restricted to live, singleton newborns.^13^ The chi-square test was used to compare the distribution of maternal characteristics in the four cohorts and trends over time; when applicable the chi-square statistic for linear trend was also calculated. Multivariable linear regression was used to estimate the magnitude of the changes in mean birthweight over time, taking into account changes in the risk factors listed above. All analyses were performed using the Stata 13.1 software.^19^

Ethical approval for studies was not required in Brazil until 1996. The 2004 and 2015 studies were each approved by the Ethics Committee of the School of Medicine and School of Physical Education, Federal University of Pelotas, and written consent was obtained from the mothers.

## Results

The numbers of liveborn singletons in the four cohorts were 5816, 5168, 4147 and 4164. Birthweight information was missing for 5 (0.09%), 17 (0.33%), 1 (0.02%) and 13 (0.31%) children, respectively.

Table 1 shows the distribution of birthweight and gestational age in the four cohorts. Preterm births increased markedly from 5.8% in 1982 to 13.8% in 2015; the prevalence of early term births (37–38 weeks’ gestation) also increased from 21.9% in 1982 to 37.5% in 2015, Conversely, full-term births (39–41 weeks) decreased substantially in the period, from 62.1% to 47.9%.

**Table 1:** Distribution of birthweight among live births, Pelotas, Southern Brazil, 1982, 1993, 2004 and 2015

Birthweight (g)	1982		1993		2004		2015	
	*n*	%	*n*	%	*n*	%	*n*	%
<1000	17	0.3	11	0.2	24	0.6	15	0.4
1000–1499	40	0.8	24	0.5	29	0.7	24	0.6
1500–1999	92	1.6	82	1.6	70	1.7	56	1.4
2000–2499	329	5.7	351	6.8	249	6.0	250	6.0
2500–2999	1361	23.4	1283	24.9	1016	24.5	960	23.1
3000–3499	2211	38.1	2040	39.6	1648	39.8	1713	41.3
3500–3999	1416	24.4	1080	21.0	912	22.0	919	22.1
≥4000	345	5.9	280	5.4	198	4.8	214	5.2
Not weighed	5		17		1		13	
<2500	8.2		9.1		9.0		8.3	
Mean birthweight in g (SD)	3201 (554)		3169 (539)		3167 (554)		3198 (537)	
Gestational age (weeks)								
<37	265	5.8	517	11.2	567	13.7	576	13.8
37–38	1007	21.9	906	19.7	1244	30.0	1562	37.5
39–41	2854	62.1	2661	57.8	2064	49.8	1996	47.9
42+	469	10.2	518	11.3	267	6.4	30	0.7
Number of children	5816		5168		4147		4164	

For consistency, we reanalysed the data from the four cohorts using only the date of the LMP to assess gestational age, instead of also using ultrasound results in the two more recent cohorts. The resulting prevalences of preterm birth were 5.8%, 11.2%, 16.2% and 17.7% in 1982, 1993, 2004 and 2015, respectively. The corresponding prevalences of early term births were 21.9%, 19.7%, 27.6% and 33.0%.

The distribution of birthweights was remarkably similar in the four cohorts, as was the case for the mean and standard deviation values. The proportion of newborns with low birthweight remained stable at around 8% to 9% throughout the period (*P* for trend = 0.80). Mean birthweight also remained stable at around 3.2 kg (*P* for trend = 0.47)

As the sources of information on gestational age varied over time, with many missing cases in 1982 and 1993, analyses of socioeconomic and skin colour inequalities are only presented for birthweight. Table 2 shows LBW prevalence according to sex, income and maternal skin colour. As expected due to the use of the same absolute cutoff for both sexes, prevalence was higher among girls than boys in the four cohorts. There was no evidence of a time trend in low birthweight for either sex. In all cohorts, LBW was most frequent in the poorest income quintile and lowest in the richest quintile, except for 2004 when prevalence in the third quintile was slightly lower than in the richest quintile. Regarding maternal skin colour, prevalence was lowest for children born to white mothers, except in 2015 when there was no statistical evidence of a difference.

**Table 2:** Prevalence (95% CI) of low birthweight according to sex of the newborn, quintiles of family income, maternal schooling and maternal skin colour, Pelotas, Southern Brazil, 1982, 1993, 2004 and 2015

Variable	Percent low birthweight by birth cohort				*P* (χ^2^ linear trend)
	1982	1993	2004	2015	
Sex	*P* = 0.003	*P* = 0.005	*P* = 0.044	*P* = 0.089	
Male	7.2 (6.3; 8.1)	8.0 (6.9; 9.0)	8.1 (7.0; 9.3)	7.6 (6.5; 8.7)	0.476
Female	9.3 (8.2; 10.4)	10.2 (9.1; 11.4)	9.9 (8.6; 11.2)	9.1 (7.8; 10.3)	0.784
Family income quintiles	*P* < 0.001	*P* = 0.001	*P* < 0.001	*P* = 0.002	
Q1	13.5 (11.5; 15.4)	10.4 (8.5; 12.3)	13.1 (10.8; 15.4)	11.7 (9.5; 13.9)	0.505
Q2	8.6 (7.0; 10.2)	10.4 (8.6; 12.1)	10.5 (10.8; 15.4)	8.0 (6.1; 9.8)	0.806
Q3	6.9 (5.4; 8.3)	9.1 (7.2; 11.0)	6.6 (4.9; 8.3)	6.8 (5.1; 8.6)	0.640
Q4	6.6 (5.1; 8.0)	9.0 (7.3; 10.8)	7.5 (5.7; 9.2)	8.4 (6.5; 10.2)	0.257
Q5	5.7 (4.4; 7.0)	6.4 (4.9; 7.9)	7.0 (5.3; 8.8)	6.7 (5.0; 8.4)	0.273
Maternal skin colour^a^	*P* = 0.007	*P* = 0.034	*P* = 0.053	*P* = 0.595	
White	7.8 (7.0; 8.5)	8.6 (7.8; 9.5)	8.5 (7.5; 9.4)	8.5 (7.5; 9.5)	0.290
Brown	10.3 (8.5; 12.1)	10.0 (6.1; 13.9)	8.7 (5.4; 11.9)	7.7 (5.4; 9.9)	0.058
Black		10.8 (8.8; 12.8)	11.0 (8.9; 13.1)	8.2 (6.1; 10.4)	

a The test for linear trend according to maternal skin colour compares white-skinned mothers against black- or brown-skinned mothers, given that in 1982 the information was collected for two categories.

The magnitude of absolute income-related inequalities was summarized by the slope index, which was equal to -8.7% points [standard error (SE) 1.3] in 1982, -4.5 (SE 1.4) in 1993, -7.6 (SE 1.6) in 2004 and -4.7 (SE 1.6) in 2015. The concentration indices for relative inequalities were equal to -16.3 (SE 2.5) in 1982, -8.9 (SE 2.5) in 1993, -13.3 (SE 3.0) in 2004 and -9.5 (SE 3.0) in 2015. Both indices show that inequalities remained unchanged during the study period.

Similar analyses for mean birthweight are shown in Table 3. Boys were about 100 g heavier than girls in all cohorts. Mean birthweight increased by 88 g among the poorest quintile (*P* for trend 0.004) and decreased by 57 g (*P *= 0.049) in the fourth and by 80 g (*P* < 0.001) in the richest quintile. The mean difference between the richest and poorest quintile fell from 249 g in 1982 to 81 g in 2015. Regarding maternal skin colour, mean birthweight remained stable for children born to white mothers, but increased by about 60 g (P = 0.01) for those born to black and brown-skinned women.

**Table 3:** Mean (95% CI) birthweight in grams according to sex of the newborn, quintiles of family income and maternal skin colour. Pelotas, Southern Brazil, 1982, 1993, 2004, and 2015

Variable	Mean birthweight (g) by birth cohort				*P* (linear trend)
	1982	1993	2004	2015	
Sex	*P* < 0.001	*P* < 0.001	*P* < 0.001	*P* < 0.001	
Male	3261 (3241; 3281)	3235 (3213; 3256)	3216 (3192; 3240)	3260 (3237; 3284)	0.509
Female	3139 (3120; 3159)	3104 (3084; 3124)	3113 (3090; 3137)	3133 (3111; 3156)	0.687
Family income	*P* < 0.001	*P* < 0.001	*P* < 0.001	*P* = 0.006	
Q1	3058 (3027; 3090)	3113 (3080; 3147)	3078 (3038; 3117)	3146 (3108; 3185)	0.004
Q2	3164 (3131; 3196)	3136 (3105; 3167)	3116 (3076; 3156)	3198 (3160; 3235)	0.428
Q3	3222 (3191; 3254)	3144 (3109; 3180)	3186 (3150; 3222)	3219 (3183; 3255)	0.996
Q4	3256 (3225; 3286)	3208 (3174; 3243)	3233 (3198; 3269)	3199 (3164; 3235)	0.049
Q5	3307 (3276; 3338)	3255 (3224; 3287)	3224 (3187; 3260)	3227 (3191; 3262)	<0.001
Maternal skin colour^a^	*P* < 0.001	*P* < 0.001	*P* = 0.001	*P* = 0.523	
White	3216 (3200; 3232)	3189 (3172; 3205)	3184 (3165; 3203)	3201 (3182; 3221)	0.122
Brown	3135 (3100; 3169)	3077 (3010; 3143)	3160 (3102; 3218)	3184 (3141; 3227)	0.010
Black		3108 (3071; 3144)	3107 (3065; 3148)	3194 (3153; 3234)	

a The test for linear trend according to maternal skin colour compares white-skinned mothers against black- or brown-skinned mothers, given that in 1982 the information was collected for two categories.

Because there were important changes in the distribution of risk and protective factors associated with birthweight (Table 4), we used multivariable linear regression to estimate the likely changes in birthweight over time, had the distribution of these factors remained constant (Table 5). In the first analyses, we adjusted for all factors in Table 4 except for diabetes and hypertension. Whereas in the unadjusted analyses mean birthweight in 2015 was only 4 g lower than in 1982, after adjustment for changes in risk factors, mean birthweight became 160 g lower in 2015 than in 1982. The effects of adjustment were larger for the richest (-213 g) than for the poorest quintile (-103 g). Further adjustment for reports of diabetes and hypertension changed the overall estimate of the reduction in birthweight over time from 160 g to 153 g [95% confidence interval (CI) 128–177].

**Table 4:** Evolution of risk and protective factors for low birthweight, 1982–2015

	Cohort			
	1982	1993	2004	2015
Family income <1 minimum wage	21.7%	18.5%	21.0%	12.7%
Not in marriage or union	8.2%	12.4%	16.4%	14.2%
Black or brown skin colour	17.9%	22.6%	26.9%	28.2%
Maternal age > = 35 years	9.8%	10.9%	13.3%	14.5%
Schooling <4 years	33.0%	27.9%	15.4%	9.2%
Maternal height <150 cm	10.9%	4.5%	6.9%	2.5%
BMI <18.5 kg/m^2^	6.6%	8.6%	5.0%	3.7%
BMI > = 30 kg/m^2^	3.7%	4.6%	6.1%	18.4%
Smoking during pregnancy	35.6%	33.2%	27.6%	16.6%
Primiparity	39.6%	35.3%	39.7%	49.4%
Parity 2 or greater	16.1%	19.4%	18.3%	8.5%
Birth interval <24 months	18.8%	11.2%	8.6%	5.8%
Antenatal care <4 visits	15.8%	11.6%	6.9%	5.6%
Report of gestational diabetes^a^	0.3%	2.8%	2.9%	8.6%
Report of hypertension in pregnancy^a^	5.3%	15.7%	23.7%	25.2%

a Due to changes in diagnostic criteria and in data collection methods, and to lower number of antenatal care visits for diagnosis of these conditions, the prevalence of diabetes and hypertension in the earlier cohorts was likely underestimated.

**Table 5:** Multivariable linear regression analyses showing differences in mean birthweight in the four cohorts with adjustment for changes over time in risk and protective factors^a^

		1982	1993	2004	2015
Unadjusted	Mean (g)	3201	3169	3166	3198
	Estimate	0 (reference)	−32	−35	−4
	95% CI		(−53; −12)	(−56; −13)	(−25; 18)
Fully adjusted (all births)	Estimate	0 (reference)	−107	−112	−160
	95% CI		(−87; −128)	(−90; −135)	(−136; −184)
Fully adjusted (poorest quintile)	Estimate	0 (reference)	−54	−84	−103
	95% CI		(−101; −7)	(−136; −32)	(−163; −44)
Fully adjusted (richest quintile)	Estimate	0 (reference)	−128	−147	−213
	95% CI		(−175; −80)	(−197; −97)	(−265; −161)

a Adjusted for family income in minimum wages, maternal skin colour, age, schooling, marital status, height, body mass index, smoking, parity, birth interval and antenatal care.

## Discussion

Our results show that preterm and early term births increased markedly over a 33-year period in the Brazilian city of Pelotas. For gestational age, changes over time are affected by differences in the methods of assessment of gestational age, and the high proportion of missing cases in 1982 and 1993 when only the date of the last menstrual period was used. Missing data were more common among less educated and poorer mothers, and thus the prevalence of preterm births was likely underestimated in the early cohorts. With increased use of ultrasound during pregnancy, our methods of gestational age assessment changed in the 2004 and 2015 cohorts. In the latter, 84.4% of all newborns had ultrasound results during the first or second trimester recorded in their mother’s antenatal cards, and the prevalence of preterm birth for these gestations was 13.8%. During the postpartum interview, 90.7% of the mothers provided information on the date of the last menstrual period, and the prevalence of preterm birth was 18.9%. Even if prevalence for gestations with missing data was twice as high for gestations with existing information as those with data, the corrected prevalence would be 7.0% in 1982 and 12.4% in 1993, which are lower values than for the more recent cohorts. The rise in preterm births was even more marked when we analysed the four cohorts using LMP data. Therefore, in spite of different methods being used in each study, there is strong evidence of an increase in preterm deliveries in Pelotas.

The increased preterm prevalence had been described in our earlier publication^8^ and is consistent with other Brazilian studies. A systematic review of peer-reviewed literature showed rising trends from the 1990s onwards, particularly in the southeastern and southern regions where Pelotas is located.^20^ In the Ribeirão Preto cohorts, preterm births increased from 7.5% in 1978–79 to 12.8% in 2004.^21^ Overmedicalization of childbirth, and in particular the sharp increase in cesarean sections, have been blamed for the current epidemic of preterm deliveries in Brazil.^22–25^ In Pelotas, cesarean sections increased from 27.7% to 65.1% of all deliveries from 1982 to 2015, when it accounted for 86.6% of all births in the richest quintile.^26^ Many caesarean sections are scheduled, typically when gestational ages are estimated to reach 38 weeks. Each week of gestational age from 37 to 40 weeks is associated with average gains of 150 g for girls and of 170 g for boys, so that even minor shifts could lead to the effect observed among wealthy women.^27^

One important finding of our study is that—for the whole population—mean birthweight and LBW prevalence remained stable. The only other Brazilian study spanning several decades was carried out in the southeastern city of Ribeirão Preto and showed an increase in LBW from 7.2% to 10.7% from 1978–79 to 1994.^21^ More recent time-series based on the National Live Birth Registration System showed a slight increase in national low birthweight prevalence from 7.9% in 1995 to 8.4% in 2015.^24^ It is paradoxical to find that the highest prevalences of low birthweight are found in the most developed regions of the country (the south and southeast), compared with the poorer regions (the northeast and north). It is postulated that this paradox is explained by excessive cesarean sections in the richest areas.^28^^,^^29^

Our results are compatible with other Brazilian studies. Prevalence of low birthweight in Latin America is estimated at 9%, with 7% in Argentina and 8% in Uruguay, countries that are closest to Pelotas.^1^

Brazilian studies on LBW suggest that the main risk factors include low family income, low education, black or brown skin colour, young maternal age, short stature, low pre-pregnancy weight, primiparity, short birth intervals, lack of prenatal care and maternal smoking during pregnancy.^30–33^ Nearly all of these risk factors evolved favourably in Pelotas from 1982 to 2015 (Table 4).^14–16^^,^^26^ Women became more educated, taller and less likely to smoke or to present with underweight (low body mass index). There are now fewer adolescent mothers, parity is lower and birth intervals longer. The number of antenatal care visits increased substantially. In light of all such changes, one would expect the prevalence of low birthweight to be reduced, and mean birthweight to increase. When we accounted for changes in risk factors over time, mean birthweight in 2015 became 160 g lower than in 1982. The difference was more marked for women in the richest quintile (213g) than in the poorest quintile (103 g). We also ran a model with the above-mentioned covariates plus maternal reports of diabetes and hypertension, for which the time-series (Table 4) was likely affected by changes in diagnostic criteria^34^ and by increased use of antenatal care with greater opportunity for diagnosis. As in the previous analyses, the mean birthweight in 2015 was substantially lower than would be expected (152 g on average) given the changes in risk and protective factors over time.

The findings from this regression analysis support the hypothesis that obstetric interventions, which increased over time and are particularly frequent among high-income women, may explain why birthweights failed to increase in accordance with the reduction in the prevalence of risk factors. A simple comparison of birthweights in vaginal and cesarean deliveries is not useful, because the latter include a mixture of procedures with medical indications (associated with lower birthweights due to morbidity) and those without medical indications (which would primarily affect women of high socioeconomic position whose newborns should present higher birthweights). We do not have comparable data on reasons for caesarean sections in the four cohorts, and in addition there are indications that obstetricians may often report medical indications for purely elective procedures; for these reasons, it was not possible to separate these two categories of caesarean sections and assess their specific associations with birthweights.

Time trends in low birthweight prevalence according to income groups were not as clear-cut as for mean birthweight, which suggests that the main impact of caesarean sections has been on babies born weighing 2500 g or more. This is consistent with the marked increase in the prevalence of early term deliveries at 37–38 weeks, from 22.3% in 1982 to 37.1% in 2015, which must have had a negative impact on mean birthweight. The hypothesis is also supported by the actual decline in mean birthweight among children born to wealthy women (Table 3), accompanied by an increase among those born to poor women, among whom the prevalence of caesarean sections is much lower.

During the 33-year period covered by our cohorts, there have been substantial improvements in maternal and child health in Brazil as a whole,^25^ which are reflected in the data from Pelotas. These positive changes, however, were not reflected in the distribution of birthweights, which remained stable in spite of marked reductions in the prevalence of its main known risk factors. The extremely high rates of caesarean sections may be held accountable for the lack of progress.

## Funding

The four cohorts received funding from the following agencies: Wellcome Trust, International Development Research Centre, World Health Organization, Overseas Development Administration of the United Kingdom, European Union, Brazilian National Support Program for Centres of Excellence (PRONEX), Brazilian National Council for Scientific and Technological Development (CNPq), Science and Technology Department (DECIT) of the Brazilian Ministry of Health, Research Support Foundation of the State of Rio Grande do Sul (FAPERGS), Brazilian Pastorate of the Child, and Brazilian Association for Collective Health (ABRASCO).

**Key Messages**

- At the population level, mean birthweight remained stable from 1982 to 2015, as did low birthweight.
- Preterm birth prevalence increased markedly, despite changes in methods of ascertainment over time.
- The stability in mean birthweight resulted from a combination of increases among children born to women in the poorest quintiles and declines in the richest quintiles.
- The prevalences of most risk factors for low birthweight were markedly reduced over time; after adjustment for these factors, it is estimated that mean birthweight declined by 160 g from 1982 to 2015.
- The most likely explanation for these results is the extremely high prevalence of caesarean section, particularly among rich women, with a resulting increase in preterm and early term deliveries due to schedule deliveries.

## Pelotas Cohorts Study Group

Aluisio J D Barros,^1^ Ana M B Menezes,^1^ Andrea Dâmaso Bertoldi,^1^ Diego G Bassani,^2^ Fernando C Wehrmeister,^1^ Helen Gonçalves,^1^ Iná S Santos,^1^ Joseph Murray,^1^ Luciana Tovo-Rodrigues,^1^ Maria Cecilia F Assunção,^1^ Marlos Rodrigues Domingues^1^ and Pedro R C Hallal^1^

^1^Federal University of Pelotas, Brazil and ^2^University of Toronto, Canada.

**Conflict of interest:** None declared.
